# *“Death is a better option than being treated like this”*: a prevalence survey and qualitative study of depression among multi-drug resistant tuberculosis in-patients

**DOI:** 10.1186/s12889-020-08986-x

**Published:** 2020-06-03

**Authors:** R. Huque, H. Elsey, F. Fieroze, J. P. Hicks, S. Huque, P. Bhawmik, I. Walker, J. Newell

**Affiliations:** 1 The ARK Foundation, Suite C-3 & C-4, House 06, Road 109, Gulshan-2, Dhaka-1212, Dhaka, Bangladesh; 2 Department of Health Sciences, University of York, Seebohm Rowntree Building, York, YO10 5DD UK; 3 Leeds Institute of Health Sciences, University of Leeds, 10.31 Worsley, Leeds, LS2 9JT UK

**Keywords:** Tuberculosis (TB), Multi-drug resistant (MDR-TB), Depression, Mental health, Co-morbidities, Bangladesh, Low and middle income countries (LMIC)

## Abstract

**Background:**

Understanding of the relationship between multi-drug resistant tuberculosis and mental health is limited. With growing prevalence of multi-drug resistant tuberculosis, addressing mental ill-health has potential to improve treatment outcomes and well-being. In several low and middle-income contexts hospitalisation during treatment is common. Understanding of the impact on mental ill-health are required to inform interventions for patients with multi-drug resistant tuberculosis.

Our aim was to identify the prevalence of comorbid depression among in-patients being treated for multi-drug resistant tuberculosis and to explore their experiences of comorbid disease and the care they received in a large specialist chest hospital in Dhaka, Bangladesh.

**Methods:**

We conducted a quantitative cross-sectional survey among 150 multi-drug resistant tuberculosis in-patients (new cases = 34%, previously treated = 66%) in 2018. A psychiatrist assessed depression was assessed with the Structured Clinical Interview for Depression (SCID DSM-IV). We used multi-level modelling to identify associations with depression. Experience Bangladeshi researchers conducted qualitative interviews with 8 patients, 4 carers, 4 health professionals and reflective notes recorded. Qualitative data was analysed thematically.

**Results:**

We found 33.8% (95% CI 26.7%; 41.7%) of patients were depressed. While more women were depressed 39.3% (95% CI 27.6%; 52.4%) than men 30.4% (95% CI 22%; 40.5%) this was not significant. After controlling for key variables only having one or more co-morbidity (adjusted odds ratio [AOR] = 2.88 [95% CI 1.13; 7.33]) and being a new rather than previously treated case (AOR = 2.33 [95% CI 1.06; 5.14]) were associated (positively) with depression. Qualitative data highlighted the isolation and despair felt by patients who described a service predominantly focused on providing medicines. Individual, familial, societal and health-care factors influenced resilience, nuanced by gender, socio-economic status and home location.

**Conclusions:**

Patients with multi-drug resistant tuberculosis are at high risk of depression, particularly those with co- and multi-morbidities. Screening for depression and psycho-social support should be integrated within routine TB services and provided throughout treatment.

## Background

Mental and physical health are inextricably linked, mutually reinforcing and can be considered a syndemic, with the synergistic interaction of these two conditions leading to an excess burden of disease [1, 2]. In this bio-directional relationship, physical health conditions increase the risk of poor mental health, which in turn undermines health-seeking behaviour, diagnosis, management and outcomes [3]. Cases of multi-drug resistant tuberculosis (MDR-TB) continue to grow globally with 160,684 cases of MDR/Rifampicin Resistant-TB detected and notified in 2017 [4]. Globally, treatment success for MDR-TB patients at 55% globally, remains much lower than for drug-sensitive TB, although several high burden countries, including Bangladesh are achieving better treatment success rates of over 70% [4]. The need to identify and successfully treat patients with MDR-TB is a global priority, and yet, the limited evidence of the relationship between mental health and MDR-TB undermines the development, testing and implementation of interventions to realise this goal [2].

There is a growing body of evidence of the prevalence of mental ill-health among those with MDR-TB. A recent large cross-sectional study of 1279 MDR-TB patients in routine care in Pakistan found 42.8% (95% CI 40.1; 45.5) to be depressed [5]. Smaller studies have found a range of levels of depression, including 16% among MDR-TB patients with co-morbid HIV infection in India [6] to 52% depressed at baseline and 13% incident cases during treatment in a review of 75 patients’ case notes following screening for psychiatric disorders in Peru [7]. It should be noted that the strong treatment required for MDR-TB itself can cause psychiatric side effects [8], with cycloserine particularly implicated [9] and these are often more debilitating than other side effects [10]. A narrative synthesis of evidence illustrates how the stigma, loss of livelihood and discrimination leads to catastrophic social and economic costs for patients [11].

For drug-sensitive TB, an analysis of World Health Survey data from across 48 low and middle-income countries (LMIC) found those with TB to have a significantly higher odds of having a depressive episode odds ratio (OR) 3.68 (95% CI 3.01; 4.50) than those without TB and this was significantly associated with worse health status [12]. The bi-directional relationship has been identified in a Korean cohort where the risk of TB was 2.63 times higher among those who were depressed [13].

While there is, as yet, limited evidence of the association, it is hypothesised that poor mental health may also trigger loss to follow up among MDR-TB patients [2]. A meta-analysis of MDR-TB patients found the median time when loss to follow-up occurred was 7 months (IQR = 3–11) with most of this loss occurring in the initial phase of treatment (75% in the first 11 months) [14].

The importance of using appropriate screening tools for depression is highlighted in a recent review of community MDR-TB programmes in Bangladesh, where despite only 5% of cases having documented psychiatric symptoms in a chart review, once 60 patients were interviewed, 60% reported psychiatric symptoms. As interviews were not conducted by a psychiatrist, it is hard to determine the clinical significance of these symptoms, however the study does indicate potential missed opportunities for mental health treatment [15]. The limited but growing evidence base has raised the profile of treatment and support for depression within National TB programmes. A recent survey of National TB Programme (NTP) directors from 26 high-burden TB countries found that while only two NTPs regularly screened for depression, 17 directors would consider integrating mental health treatment into national TB guidelines and services [16]. Current World Health Organisation (WHO) guidance on MDR-TB treatment emphasises care and support for MDR-TB patients, with psychological support specifically mentioned, however no further details of what this should include or how this can be delivered in different MDR-TB treatment settings are provided [17]. Previous work on providing psycho-social support in MDR-TB services has highlighted feasibility challenges [18] and understanding of the content and modalities of mental health interventions for MDR-TB patients are limited. Despite an increasing focus on out-patient care for MDR-TB [17], in many high-burden countries, patients are still hospitalised particularly for the intensive phase of treatment. In light of this, we wished to understand the mental health experience of MDR-TB patients that are treated as in-patients to inform the development of subsequent interventions to improve care and treatment.

## Methods

Our aim was to identify the prevalence of comorbid depression among in-patients being treated for MDR-TB, as well as the socio-demographic and health related correlates of comorbid depression, and to explore patient experiences of comorbid disease and the care they received.

### Design

We used a sequential mixed-methods design [19] with an initial quantitative cross-sectional survey that informed the design of qualitative interviews, reflective researcher notes and the codes developed during analysis of the qualitative data.

### Setting

The study was set in the largest tertiary government run chest hospital in Dhaka, Bangladesh treating lung diseases and acting as the largest referral hospital for TB. At the time of the study (January to July 2018), the hospital had 73 beds for male TB (both MDR- and extensively drug-resistant XDR-TB) patients and 46 for female patients in separate male and female wards. Due to limited space, the female ward often includes non-TB patients. There was one doctor for the male and female wards, six nurses for each ward during the day-shift and three nurses providing the night-shift. Patients are referred from across Bangladesh normally following confirmation of rifampicin resistance. Following further drug susceptibility testing at the hospital, MDR-TB patients will normally spend the first month of their treatment in the hospital before being discharged following two successive smear-negative results. They will then be managed by TB services in the community. Those that remain smear positive will stay in the hospital and have a further drug susceptibility test at 3 months.

### Participants

For the cross-sectional survey our participants were adult in-patients diagnosed with MDR-TB. In close consultation with hospital staff, we agreed to exclude patients who were under 18 years old, had extensive drug resistance, were due to leave hospital within a week or were deemed too ill by the health professionals or researchers to take part in the study.

Through the qualitative interviews we aimed to understand the impact of MDR-TB on patient’s lives and their experiences of treatment in the hospital. To deepen our understanding of these issues we purposively sampled in-patients and cured patients, carers and health professionals. We aimed to interview both male and female patients and carers to understand gender differences in care and experiences.

### Sample size

Given the limited information on depression in this patient group we conservatively estimated the proportion of people with depression to be 50%, and we therefore required a sample size of 97 to estimate this prevalence with 95% confidence intervals of at most +/− 10 percentage points. For the cross-sectional survey, based on in-patient flow at the hospital and our exclusion criteria, we estimated that approaching all in-patients over a six-month period would meet our sample size target.

To determine the size of the qualitative sample, we drew on principles of ‘information power’ [20]: our aim was relatively broad, attempting to understand impacts of MDR-TB on mental health among all patients, so in recognition of this we included patients of different ages, male and female; we also included care-givers to provide perspectives on their family-member’s experiences. To enhance information-power we drew on theory to analyse our qualitative data in the form of the socio-ecological model [21] which identifies relationships at individual, relationship, societal, institutional and policy levels. Health professionals and policy makers provided insights into the institutional and policy responses to mental ill-health. We planned to interview four male and four female patients of different ages. Conscious of the poor physical and mental health of many patients, we planned to conduct multiple interviews with the same patient if a single long interview was not possible. We planned to interview four carers and four health professionals.

### Cross-sectional survey measures

An initial questionnaire was conducted with recruited patients once informed consent had been taken. This included socio-demographic information, self-reported co-morbidities and substance use, including mental ill-health, any treatment taken for mental ill-health and MDR treatment side-effects. Participants were asked to report separately their use of tobacco, alcohol or any other substance and to state if they had any other health conditions. For those patients who self-reported a co-morbidity, following patient consent and health professional approval, their patient records were checked by the researchers and the nature of the comorbidity was recorded. This socio-demographic questionnaire was conducted face-to-face in Benagli by researchers who were all employed by a Bangladesh research organisation, The ARK Foundation. Within two-weeks of this initial assessment, a psychiatrist working as part of our research team undertook a clinical assessment with these patients using the depression module of the Bengali version of the Structured Clinical Interview for DSM-IV (SCID) [22]. The SCID is a well-used tool for psychiatric assessments and has been used in previous studies in Bangladesh [23, 24]. In our study, the depression module of the research version of this tool was used. During the assessment, any patient identified as being severely depressed or having suicidal thoughts was referred to their hospital clinician.

Qualitative individual interviews and reflective notes were used following the initial analysis of the cross-sectional survey. Topics explored in the interview guides were informed by the preliminary analysis of the questionnaire data and included: their experience of becoming ill, diagnosis and care; following diagnosis with MDR-TB, reactions and subsequent relationships with family members, friends and wider community, experiences of hospitalisation and treatment, understanding and experiences of mental ill-health and recommendations for improving the support to patients. Interview guides for carers covered similar topic areas in relation to the patient they were attending and their perspectives of their mental health and care in the hospital. Cured patients were asked to reflect on their experiences throughout and after completion of treatment. Provider interviews focused on the current service provision, their perceptions of patient mental well-being and their confidence to support patients with mental ill-health.

Interviews were conducted by one senior female and one early-career female qualitative researcher in Bengali, employed by the ARK Foundation. Interviews were conducted in the ward or adjoining outside area, as preferred by the participant. Following identification by a health care provider, cured patients were interviewed over the phone. Health care providers were interviewed in their offices in the hospital or their private practices. All interviews were audio-recorded and then as soon as possible, transcribed into English. Both researchers spent considerable time at the hospital building rapport with patients, carers and staff. They recorded their observations and reflections on the physical, social and clinical environment. Thematic qualitative analysis [25] was conducted using the transcripts and the reflective notes of the researchers. After initial familiarisation with all the data, a coding frame was developed jointly by FF and senior researcher (HE). FF coded all transcripts and reflective notes. HE double-coded 5 transcripts to compare the use of codes and to further refine the coding framework.

### Statistical analysis

We calculated appropriate descriptive statistics for the socio-demographic and key health related variables to describe our samples key relevant characteristics. As per the SCID diagnoses we estimated the prevalence of any level of depression and the prevalence of mild, moderate and severe depression separately, along with the 95% confidence intervals (CI) for each prevalence estimate calculated via the Wilson score method [26]. We then used a logistic regression model to estimate the associations between a range of potentially important independent variables and the binary outcome of depressed/not depressed as per each patients’ SCID diagnosis. We selected the independent variables included in the model based on those variables that were identified as likely to be important in causing and/or influencing depression in MDR-TB patients from the literature, and we did not do any further model selection. We calculated adjusted odds ratios (AOR) from our logistic regression model coefficients along with their 95% (Wald-based) confidence intervals. We excluded any patients from the model who had any missing outcome or independent variable data. We verified that none of the logistic regression model assumptions were violated using appropriate methods (e.g. residual plots) [27]. We analysed all data using R statistical software (version 3.5.1) [28, 29].

### Ethics

Ethics approval was given by School of Medicine Ethics Committee, University of Leeds (Ref no: MREC16–163) and the National Research Ethics Committee, Bangladesh Medical Research Council (Ref: BMRC/NREC/2016–2019/211). Informed consent was obtained from patients before the SCID assessment and from patients, carers, health professionals and policy makers before the qualitative interviews through reading out an information sheet, encouraging any questions to be asked and signing a consent sheet.

### Funding

This study was conducted as part of the research programme consortium COMDIS-HSD, which was funded by UK Aid from the UK government. The funders had no role in study design, data collection and analysis, decision to publish, or preparation of the manuscript.

## Results

We approached all 254 in-patients admitted between January and July 2018. Of these 254 in-patients, 66 did not meet our inclusion criteria as either under 18 years old (*n* = 12), being discharged within a week (*n* = 23), diagnosed with XDR TB (*n* = 5) or too ill to be interviewed (*n* = 26). Of the 188 eligible, 38 declined to be interviewed and 150 completed the initial demographic questionnaire (Table 1). Of these 150, two patients left hospital (without being discharged) and were therefore not assessed by the psychiatrist using the SCID tool [22].

**Table 1:** Participant characteristics

Variable		Mean/%	Range/n
Age (mean)		35.28	18–80
Sex	Male	62%	93
	Female	38%	57
Occupation	Student	7%	11
	Farmer	11%	16
	Labourer/factory/rickshaw driver/business	64%	96
	Domestic servant	8%	12
	Office-based (public or private)	2%	3
	Unemployed	8%	12
Marital status	Single – never married	21%	32
	Married	75%	113
	Divorced/widowed	4%	5
Education	No schooling	29%	44
	Primary/Junior	39%	59
	Secondary (to grade 10)/higher	31%	47
Living location	Rural	46%	69
	Urban	54%	81
Previously treated or new case	New MDR-TB case	34%	51
	Previously treated	66%	99
Substance use	Any substance use	50%	75
	No substance use	50%	75
Type of substance used (*N* = 75)	Current smoker	69%	52
	Drug use	9%	7
	Alcohol use	1%	1
	Smokeless tobacco	13%	10
	Multiple substance use	8%	5
History of mental disorder	Self-reported ‘yes’ to question: any history of mental disorder?	7%	10
	Self-reported ‘no’ to question: any history of mental disorder?	93%	140
Co-morbidity	Self-reported ‘yes’ to question: any co-morbidities?	27%	40
	Self-reported ‘no’ to question: any co-morbidities?	73%	110
Type of co-morbidity (*N* = 40)	Diabetes	50%	20
	Cardiac disease	13%	5
	High or low blood pressure	28%	11
	Asthma	5%	2
	Hernia	3%	1
	Breast lump	3%	1
	Kidney disease	3%	1

A total of 33.8% (95% CI: 26.7, 41.7%) of patients were diagnosed as depressed (with either a mild, moderate or severe level of depression) via the SCID tool, with most patients diagnosed with either mild or moderate depression (Table 2). Our logistic regression model only identified two variables that were statistically significantly associated with depression in our sample: patients with one or more co-morbidities had 2.88 (95% CI: 1.13, 7.33) times the odds of being diagnosed with depression compared to patients with no co-morbidities and patients who were new MDR-TB cases had 2.33 (95% CI: 1.06, 5.14) times the odds of being diagnosed with depression compared to previously treated MDR-TB cases (Table 3) .

**Table 2:** Prevalence of depression by severity as measured by SCID

Outcome	***n/N***	% (95% CI)
**All Patients**		
Any depression (mild, moderate or severe)	50/148	33.8% (26.7, 41.7)
Mild depression	27/148	18.2% (12.9, 25.2)
Moderate depression	19/148	12.8% (8.4, 19.2)
Severe depression	4/148	2.7% (1.1, 6.7)
**Males only**		
Any depression (mild, moderate or severe)	28/92	30.4% (22, 40.5)
Mild depression	17/92	18.5% (11.9, 27.6)
Moderate depression	9/92	9.8% (5.2, 17.6)
Severe depression	2/92	2.2% (0.6, 7.6)
**Females only**		
Any depression (mild, moderate or severe)	22/56	39.3% (27.6, 52.4)
Mild depression	10/56	17.9% (10, 29.8)
Moderate depression	10/56	17.9% (10, 29.8)
Severe depression	2/56	3.6% (1, 12.1)

*N* = 148 (SCID outcomes missing for 2 patients). 95% CIs calculated using Wilson score method using R package binom [28]

**Table 3:** Logistic regression exploring associations between socio-demographic and health related variables and a SCID-based diagnosis of depression

Covariates	Adjusted odds ratio (95% CI)
Age ≤ 40	Ref
Age > 40	0.95 (0.38, 2.35)
Male	Ref
Female	1.57 (0.57, 4.33)
Not currently/never married	Ref
Married	2.89 (1, 8.37)
No education	Ref
Primary/Junior	0.73 (0.29, 1.88)
Secondary/Higher	0.81 (0.29, 2.27)
Urban location	Ref
Rural location	1.19 (0.55, 2.57)
Days since treatment start^a^	1.01 (0.99, 1.02)
Previously treated MDR-TB case	Ref
New MDR-TB case	2.33 (1.06, 5.14)
Non-smoker	Ref
Smoker	1 (0.37, 2.73)
No co-morbidities	Ref
One or more co-morbidity	2.88 (1.13, 7.33)
Sought treatment for side effects	Ref
Not sought treatment for side effects	1.38 (0.63, 3.02)

*Ref* reference/comparison group for dummy coded categorical variable comparisons. *N* = 148 (SCID outcomes missing for 2 patients). Adjusted odds ratios are adjusted for all variables/levels listed in the table in a logistic regression model. 95% CIs are approximate Wald-based CIs. ^a^ Days since treatment start and SCID diagnosis: numerical discrete variable (range = 3–138)

The qualitative findings provide insights into the interlinking factors which are driving the challenges facing patients with MDR-TB including those presented above in the quantitative analysis, but also provide further depth in understanding poor mental health among patients. The characteristics of the qualitative participants are presented in Table 4. The socio-ecological model [21] was used within the analysis to show how these factors operate at an individual, relational, societal, institutional and policy level. These headings are used to structure the qualitative findings below.

**Table 4:** Qualitative interview participant characteristics

Care-givers							
Participant ID	Age	Relationship with patient	Education	Home location	Marital status	Occupation	Sex of patient
CGFP01	31–50	Mother	No formal education	Rural	Married	Homemaker	Female
CGFP02	31–50	Mother	Primary education	Rural	Married	Homemaker	Female
CGMP01	31–50	Mother	No formal education	Rural	Married	Homemaker	Male
CGMP02	18–30	Wife	Secondary education	Rural	Married	Homemaker	Male
**Patients, cured and on treatment**							
Participant ID	Age	Sex	Education	Home location	Marital status	Occupation	
CPF01	18–30	Female	Secondary education	Rural	Widow	Unemployed	
CPF02	18–30	Female	Higher secondary education	Urban	Unmarried	Student	
CPM01	18–30	Male	Higher secondary education	Urban	Married	Small business	
CPM02	31–50	Male	Higher secondary education	Rural	Married	Small business	
TBPF01	18–30	Female	Secondary education	Urban	Married	Factory worker	
TBPF02	18–30	Female	Primary education	Rural	Married	Home-maker	
TBPF03	18–30	Female	Primary education	Urban	Married	Home-maker	
TBPF04	51 plus	Female	Secondary education	Rural	Married	Home-maker	
TBPM01	18–30	Male	Primary education	Rural	Married	Garment worker	
TBPM02	18–30	Male	No education	Rural	Unmarried	Garment worker	
TBPM03	31–50	Male	Secondary education	Urban	Married	Small-business	
TBPM04	18–30	Male	Unknown	Rural	Married	Unemployed	
**Health Professionals**							
Participant ID	Age	Sex	Education		Occupation		
DR01	31–50	Male	MBBS		Doctor		
DR02	31–50	Male	MBBS		Doctor		
N01	31–50	Female	Diploma in Nursing		Nurse		
N02	31–50	Female	Diploma in Nursing		Nurse		
**Policy Makers**							
Participant ID	Age	Sex	Education		Occupation		
PM01	60	Male	MBBS, MPhil		Government senior official/academic		
PM02	55	Male	MBBS, MCPS, FCPS, MD, FCCP		Government senior official		
PM03	45	Female	MBBS, MPH		Government senor official		

### Individual level

#### Dealing with side-effects

The majority of the 8 patients interviewed described experiencing one or more of the known side effects of their medication including vomiting, headaches, blurred vision, aching joints, hearing loss and imbalance. The impact on mood was also clear:

*“Like yesterday … I was planning not to take the medicines. Why? Because, I cannot tolerate it anymore and my mentality has been in such distress that I wanted to skip the day’s treatment.” (TBPM04, MDR-TB patient, male)*

Health professionals were very conscious of the impact of these side effects on patients’ mental well-being and how this could influence their commitment to continuing treatment:

*“When these situations (side effects) occur during the first three or four weeks of treatment the patients get quite depressed. They think, ‘I am taking so many medicines; but still my physical condition is worsening day by day’ … . There are incidents of patients who abscond from the hospital. One of the major challenges we face is when the patient stops taking treatment. Sometimes the patients think that the treatment will not work for him or her because of their depression. What happens is that the patient may receive the medicines everyday but not take them and throw them away somewhere. I think, this is one kind of expression of depression of the patient.” (DR01, Hospital doctor, male)*

#### Continuing low mood after treatment completion

The feelings of low mood were identified by many, including the health providers, as a continual experience, not just linked to taking the medication. The interviews with cured patients highlighted how, for some, even after being successfully treated the feelings did not subside:

*“The things that depressed me before are still depressing me now. Such as, I couldn’t finish my study, which is very sad for me. Secondly, this was not for a year or two, this was five years. For these five year of gap, I have lost many things in life. Compared to other girls, in life I am sacrificing a lot of things. These things still hurt me mentally. If I tell anyone, they will ask me to forget everything. It is easier to ask to forget, but difficult to do. That is why I don’t tell anyone. I just stay by myself. I am just waiting to see how long this depression will last.” (CPF01, Cured MDR-TB patient, female)*

#### Diagnosis and dealing with multiple health problems

Many patients described a complex route to TB diagnosis, seeking diagnosis in a range of private and public facilities. Many were unclear regarding their diagnosis and were not specific regarding other health conditions that they faced. However, what was clear was that these multiple diagnoses compounded the worry felt by patients. This young, married patient describes how she became ill following the birth of her child:

*“I went to the TB-centre at the Upazila [sub-district health centre]. There, I underwent three diagnostic tests, but my disease was not diagnosed. After that I went to see a doctor of [diabetes clinic] who also treats patients here. There, I had diagnostic tests for my whole body. The doctor said that I have liver problem, jaundice, anaemia & some chest problems. Then, I was in doubt. I went to a private hospital & did an x-ray. With that x-ray & other diagnostic reports, I went to BRAC [and was referred to this hospital] … I am still taking the drugs and I started to feel ok. But now, another problem has occurred, I have a breast-tumour. Now a specimen will be taken today for diagnosis. Right now, my mind is (laughter) ok; but deep inside I am worried about today’s diagnostic test. I am already taking one sort of medication right now; but now I am having another health problem. Who knows what will happen next? Thinking about it, I am stressed right now.” (TBF03, MDR-TB patient, female)*

#### Building personal resilience

The patient interviews highlighted the different ways patients found to take their mind off their condition through talking, singing, playing board games, and for those who had mobile phones, listening to music and looking at photos. Some male participants, particularly those with higher levels of education, also talked about writing diaries and songs:

*“The truth is, whenever I feel tense, I sit down with my paper and pen. That’s the time I feel I want to write. I want to write in an effective way so that I can tell each and every person in Bangladesh about this disease. Sir I want to write in such way so that I can express my feelings to well-known lawyers and doctors through my song or through my words. Then this disease can be beaten completely. Otherwise all the young people, my children will be destroyed.” (TBM03, MDR-TB patient, male)*

For others, religion proved a powerful source of hope and reassurance:

*“My son says his prayers five times a day, he prays to his Lord. That can give him mental peace.” (CGMP01, Care-giver, mother of MDR-TB patient)*

*“I overcame my depression praying to Allah by saying, ‘Allah I have lost everyone, but you are my hope’. I had this faith in Allah.” (CPM01, Cured MDR-TB patient, male)*

The role of information and knowledge gained either from health professionals, or more commonly from their own experience or talking to other patients, was crucial in helping patients to keep the impact of the side-effects in perspective. Understanding that these were side-effects of the medicines, rather than symptoms of their illness helped some patients to find ways to cope:

*“Towards the beginning I used to consume the medicines in the morning. I noticed that I would feel bad the entire day. I would feel demotivated to shower or to go grocery shopping. As a result, I changed my routine to take them in the afternoon. I’d take a nap or rest for a bit. Then towards the evening I would start to feel better again. It takes about four to five hours after taking the medications for me to start feeling better.” (TBPM01, MDR-TB patient, male)*

### Relationships and family

The relationships with those around them and with family members were key to patient’s ability to cope with the physical and mental challenges they faced. There were considerable differences based on gender, age and whether they had had to relocate away from their families.

#### Spousal support

Interviews with male patients and their wives demonstrated the commitment of wives to caring for their husbands. The researchers’ observations indicate how these differences impacted on the day-to-day welfare of patients as well as their mental health. For example, the male ward was frequently filled with female carers cooking, cleaning for and supporting their patients, whereas the female ward had fewer carers and while some had stoves for cooking few had the energy to buy groceries and cook for themselves. Wives commitment to their husband’s health was frequently expressed:

*“After he was admitted here, my husband told me not to go near him. I didn’t pay much attention to his instructions. Let me become infected. I told him if I contract the disease then we’ll die together. But I will not succumb to the idea that I cannot feed him and take care of him just because I may be infected as well.” (CGFP02, Care-giver, mother of MDR-TB patient)*

And as this husband explains about his wife:

*“She says, “You see, today you are suffering from this disease, but don’t be scared of it. Someday everyone will die. I will never ever leave you. It doesn’t matter what disease you get or how contaminating it is. I am here with you and I will always be.” Yes, she says so and she stays with me and this gives me positive energy.” (TBPM03, MDR-TB patient, male)*

For the wives, even when husbands were supportive, the wives themselves encouraged them not to visit, but instead to support the family at home:

*“My husband supports me. I request him not to come here; because there is no use of coming here. I am taking the medicines and I will be cured. If he remains seated beside me, will my problems be solved? `Instead, if he works.. I have a child and there are some expenses for my child, my husband also has some expenses. My family cannot bear all his expenses; he cannot remain seated beside me.” (TBPF03, MDR-TB patient, female)*

All the carers of female patients that we were able to interview were mothers of the patient. Where these women were bread-winners for their families they struggled to bear the opportunity cost of coming to care for their daughters. When husbands abandoned wives, it fell to the mother to provide care:

*“No, he [the husband] didn't take care of her. I have bought her here; he came to see her only one day. Then I told him: "son you married her after seeing we are poor, now we can't give medicines to our daughter, give us some money so we can give her medicines." He replied, “you always want money". So, that's okay, we don't want his money! Now if we can help her or not, that is our only concern.” (CGFP01, Care-giver, mother of MDR-TB patient)*

The challenges of relocating to hospital and being far from children was frequently raised by the female patients and the break in this relationship, concerns for how their children would be cared for in their absence, coupled with their fear of infecting family members further fuelled their anxiety and lowered mood:

*“She says she doesn't want to stay here: “I have two children. I want to go to the village. I don't want to stay in Dhaka.” As I told you, she feels bad for her children. She can't take care of her children. She can't feed them. Her son cries. He calls her. Then she feels sad. People strike her son. With that tension, she feels sad.” (CGFP02, Care-giver, mother of MDR-TB patient)*

Male patients’ concerns frequently focused on the livelihood of their families and feelings of guilt and worry that they were unable to provide for them:

*“Whenever I ask him, he just replies that he is worried about his family, his children and his home. He feels bad that he contracted this disease at such an early age. He stays sad thinking about these things.” (CGMP02, Care-giver wife of MDR-TB patient)*

Those with no family support were clearly identified as facing particular challenges, both in terms of loneliness and low mood, but also in eating healthily and caring for themselves:

*“For instance, I’ve noticed that some patients do not have any family members who visit them. There are two or three patients here who are completely alone. That’s one thing they suffer from, loneliness. We have family here and they don’t. They see this and they feel this absence, they feel the loneliness. Some do not have enough money. Here we are told that the government will take care of everything. But except for the rice, the rest of the food they provide - the vegetable, meat, fish at night - are inedible. As a result, we have to spend money. For some patients they do not receive any monetary help from their families.” (TBPM01, MDR-TB patient, male).*

### Societal Reponses

#### Social stigma of TB

Within the interviews, participants often struggled to talk about the levels of stigma they experienced. Frequently, at the start of the interview they would deny experiencing, but by the end many shared their stories of the negative reactions of their extended families and wider society. This female patient explains how the stigma from wider society can lead relatives and husbands to abandon their wives:

*“When I came out of my room [at home], nobody stayed around. Everybody went inside their room. When I put my washed clothes to dry, they moved my clothes away. After facing these things for many days, I said to them, “This disease affected me, so you are doing these things to me. Just think, if you had this disease.” After saying this, some people kept contact with me and some people did not. When, I was first admitted to hospital, my cousins came. After that, nobody came. And now, no one comes to me. … All the in-laws are (laughter) different kinds of people. Despite of my condition my husband kept me; so now others fight with him. They ask him why he kept a diseased wife, they also say that he will catch this disease someday.” (TBPF02, MDR-TB patient, female)*

When asked what might be driving the level of stigma within society, many identified infection and lack of knowledge about TB treatment. One patient also identified the perceptions of risk behaviours of patients:

*“People think they have got their disease from their addiction to cigarettes, tobacco or other drugs. Because of this they consider us differently from their heart.” (TBPM03, MDR-TB patient, male)*

The impact of the societal view of TB, and particularly MDR-TB were clear:

*“Sometimes I feel like I will go to [the doctor] and say, “Sir, living our life may have some significance for our family members; but we do not have any value in the eyes of other people.” You can only provide us with treatment … So, it is better to just to kill us. I said, “Please give an injection to kill me. We are not able to stay in this society. So, kill us.” (TBPF02, MDR-TB patient, female)*

Patients had the option to leave the ward to buy food in the stalls near to the hospital, however this brought its own challenges:

*“I don’t tell them [shop-keepers] about my condition. But if I wear a mask then they do not let me shop or even serve food* … *I feel terrible. I can’t even go out and get a haircut or get a shave. If we go out to nearby shops for buying groceries, then we have to either take the mask off or they won’t allow us to get close to them … It seems like death is a better option than being treated like this.” (TBPM03, MDR-TB patient, male)*

#### Peer support

Despite the challenges of living away from home as an in-patient, the majority of patients, male and female, shared positive experiences of the peer support among patients on the ward. One young female patient described how she and an older patient supported each other through times of low mood and optimism:

*“But after a while, she went into depression like me. She didn’t eat or do anything. But then, some of my medical reports started coming good. I was about to be released. So, then I told her: “Grandma, we had the same situation, we both felt depressed, that is why my reports weren’t coming good. Then I made her understood. It seemed like she was listening to me. She was also staying cheerful like me, eating food. Since all of us patients were staying together like a family, so if one person was told, others also listened and followed. So, their conditions improved too.” (CPF02, cured MDR-TB patient, female)*

Patients and carers described patients chatting, singing songs and supporting each other, as explained by this wife caring for her husband:

*“Sometimes we see that one [patient] is singing and another joins in with another song in the middle. Then laughter breaks out among them. That helps them to feel relaxed and happy.” (CGFP02, Care-giver, mother of MDR-TB patient)*

For those with limited support from relatives and friends outside the hospital this was particularly important, as one cured patient recalls:

*“I had good relations with the other patients. Since I was away from my own family, they were like family to me.” (CPF01, Cured MDR-TB patient, female)*

The close connections established between patients meant that seeing each other’s suffering and even death, was particularly affecting, as this cured patient remembers:

*“Many have died in front of my eyes in the ward. I used to see many people die from the ward beside ours. By seeing these people, I have known … I felt more broken mentally.” (CPM02, Cured MDR-TB patient, male)*

### Institutional

The interviews with doctors and nurses showed that while they recognised that many patients would be depressed throughout their stay in hospital, only a very small minority would require treatment for depression:

*“It is difficult to say this overall, but about 1% or 2% of the patients may need treatment for depression. The patient generally stays more or less depressed through the entire period of the treatment starting from when the patient was first diagnosed with MDR-TB.” (DR01, Hospital doctor, male)*

Given that 33.8% of patients were found to be depressed on the SCID assessment tool, the assumption that only 1–2% of patients required treatment is a significant underestimation. This is reflected in the responses from patients, who frequently expressed the view that health professionals were not providing any support beyond the delivery of the MDR-TB treatment:

*“If they came to us and spoke to us about our updates that would help. But they seldom come for that purpose. They visit only to administer medicine and injection., they never visit us to ask us about our health, whether we are feeling better or worse.” (TBPM01, MDR-TB patient, male)*

Several patients went further in their criticisms of staff, including the cleaning staff.

*“Even the doctors and nurses, even the cleaning staff hate us very much. Today, when we were going for sputum-specimen, the staff were also walking along [near us]. They yelled, “Hey, why don’t you walk through on the other side?” … “MDR patients are barbarians, they do not understand that their disease spreads.” … The staff hate us; so why do they come to work in this hospital? They know that there are patients here, and they say heartless words in front of us; don’t they understand we feel bad?” (TBPF02, MDR-TB patient, female)*

The researchers’ observations noted the reluctance of cleaning staff to clean the female bathrooms, feeling that this should be done by the women themselves. The male bathrooms were observed to be cleaner, whether this was due to the cleaning staff or the patients’ female care-givers, the researchers were unsure.

The lack of any means of keeping occupied was a challenge for patients and seemed to fuel their low mood.

“*We have nothing here. One day I was singing, using these bowls as musical instruments, “Bondi achi bokkhobidhi hashpatal e”. I wrote that song, but when the security guard heard it he came and attacked me. He asked me “Why are you singing?” I replied, “Sir I don’t feel good that’s why I am singing.” Then he said “No, you are not allowed to sing here. I will file a complaint against you and will take you from here.” All of us stay here like this for the whole day and night. We are like unemployed people with nothing to do. Our mood keeps swinging with the passing of time.” (TBPM03, MDR-TB patient, male)*

Interviews with doctors and nurses, coupled with the observations of the researchers pointed to a lack of infection control procedures and staff mentioned that one nurse was recently infected with MDR-TB.

At the time of the study, the hospital was beginning to phase in the new nine-month treatment regimen. Policy makers and clinicians were hopeful that this regimen would reduce the suffering and wider impacts on patient’s livelihood and depression.

## Discussion

We found 33.8% (95% CI: 26.7, 41.7) of patients with MDR-TB were depressed, 2.7% (95% CI: 1.1, 6.7) with severe depression, 12.8% (95% CI: 8.4, 19.2) with moderate depression and 18.2% (95% CI: 12.9, 25.2) with mild depression. The prevalence of depression among this patient group is much higher than that found in the general Bangladesh population, estimated at 4.1% [30]. Our findings are similar to those from a recent assessment of MDR-TB patients in Pakistan using the PHQ-9 screening tool where 42.8% (95% CI: 40.1, 45.5) were found to be depressed at the lower cut-off of 6, and 16.2% (95% CI: 14.3, 18.3) at a cut-off of 10 on the PHQ-9 [5]. An area for further exploration is the extent to which patients continue to experience depression even after successful treatment for their TB, as indicated within our qualitative findings, particularly in relation to the changes in their family and social situation, occupation and experiences of stigma and self-worth.

We also found that having one or more co-morbidities (compared to none) and being a new MDR-TB (rather than a previously treated case) were positively associated with the odds of being depressed. Given that our study was powered to identify the prevalence of depression to a modest precision only and not to identify correlates of depression these results are strictly exploratory, and it is very unlikely that we have identified all important variables that are associated with depression in this patient group. The assessment of depression in the Pakistan MDR-TB population did not find significant associations with new as opposed to previously treated cases or co-morbidity [5].

The association found between mental ill-health and multiple-morbidities has been found in other studies. For example, a recent meta-analysis identified the risk for depressive disorder was twice as great for people with multimorbidity compared to those without multimorbidity RR: 2.13 (95% CI 1.62–2.80) [31]. The evidence base on the links between mental health and TB, particularly MDR-TB is still limited. However, few studies have explored the relationship between mental health and TB and other morbidities. This focus on multi-morbidities is an area where further research is needed. The positive and possibly counter-intuitive association between being a new vs a previously treated MDR-TB case and likelihood of being depressed might be explained by survivorship bias. Specifically, previously treated cases may include a larger proportion of patients that have a more resilient mental state than is typical because they were able to survive (literally or in terms of enduring treatment) previous TB treatment in part due to their greater mental resilience than those who are more prone to mental ill-health and may have been more likely to die or be lost to the health system.

Women are at greater risk of depression in the population [30], and specifically among those with drug-sensitive TB, adjusted prevalence ratio (APR) 1.23 (95% CI 1.18 to 1.27) [32] and MDR-TB, OR 1.90 (95% CI 1.52–2.38) [5]. While we found higher levels of depression among women than men, these were not significant in our adjusted model. This may be due to our lack of power to find associations within sub-groups. However, the qualitative evidence points to the societal and relational challenges facing women resulting in lower levels of social support during their treatment. Low social support has been found to be associated with depression in drug-sensitive TB patients in Ethiopia, APR 0.89 (95% CI 0.85 to 0.93) [32] and in a study of hospitalised MDR-TB patients in Nigeria, patients who were supported by their own families reported fewer psychosocial concerns compared with those who were not [33].

Our study highlights how practices within the hospital, such as not allowing male relatives to attend to female patients in the female ward and the expectation that female patients should clean their own bathrooms helped to exacerbate the limited support, both social and practical, experienced by female patients. Interviews with patients and carers illustrated how these practices are built on wider gender norms, for example, with female patients actively encouraging spouses to maintain livelihoods and care of the household rather than provide social support to the hospitalised spouse. Previous studies have provided good evidence for models of care that do not rely on hospitalisation of patients, finding ambulatory home-based care as more cost-effective [34] and preferable to patients [35] than hospitalisation. This is also advised within WHO guidelines [17].

Our mixed-methods approach highlights how health professionals may underestimate the proportion of patients that are depressed with their estimates of those needing treatment being way below the one third of patients identified as depressed. The mechanisms linking MDR-TB and depression appear to be driven by the extreme seriousness of the condition, a factor that has been identified in other chronic conditions [36], the wider impacts on the patients’ life and livelihood [6, 7, 11, 32] as well as the side-effects of drugs for MDR-TB treatment [8–10]. The impact of this is seen on both patient well-being and adherence to MDR-TB treatments [37]. The need for routine screening and provision of treatment for depression throughout the period of treatment for MDR-TB patients and integrated into routine TB care is clear from our study in Bangladesh and similar work in other LMIC settings [5, 33, 35].

A strength of our study is the use of an assessment by a psychiatrist using the SCID tool to identify depression. Given the limited existing evidence of the validity of screening tools for depression in this population groups, psychiatrist assessment can be considered the gold standard in assessment of depression.

The study has several limitations: firstly, the study was conducted in only one hospital in Bangladesh, limiting the generalisability of the findings. Secondly, our sample size limited the assessment of which factors influence or are associated with depression. Our qualitative interviews were conducted within the hospital wards, and researchers reflected that this may have undermined the ability of some participants to speak openly about their experiences and conditions. We found it harder to recruit certain participants. This was particularly noted for younger men, who may have been concerned about sharing ‘risk’ behaviours and older women, particularly those who were illiterate and may not have felt comfortable talking in-depth with researchers.

## Conclusions

A high proportion of MDR-TB patients have depression during their treatment. Those with multiple-morbidities are particularly at risk of depression. Being an in-patient with limited psycho-social support from family members or health professionals appears to exacerbate poor mental health. Improving conditions for in-patients and developing and testing interventions to support mental well-being within routine TB care should be considered.

## Data Availability

The quantitative datasets generated and analysed during the current study are available in the repository: Research Data Leeds Repository https://archive.researchdata.leeds.ac.uk/ and can be found here: https://doi.org/10.5518/754 The qualitative datasets generated and analysed during the current study are available from the corresponding author on reasonable request.

## Abbreviations

AOR: Adjusted odds ratios
APR: Adjusted prevalence ratio
CI: Confidence interval
FCCP: Fellow of the College of Chest Physicians
FCPS: Fellow of the college of physicians and surgeons
HIV: Human Immunodeficiency Virus
LMIC: Low- and middle-income country
MPhil: Master in Philosophy
MBBS: Bachelor of Medicine, Bachelor of Surgery
MD: Doctor of Medicine
MCPS: Member of the college of physicians and surgeons (Bangladesh)
MDR-TB: Multi-drug resistant tuberculosis
OR: Odds ratio
RR: Relative risk
SCID DSM-IV: Structured Clinical Interview for Depression
TB: Tuberculosis
WHO: World Health Organisation
XDR-TB: Extensively drug-resistant tuberculosis

## References

[1] Singer M, Clair S. Syndemics and Public Health: Reconceptualizing Disease in Bio-Social Context Medical Anthropology Quarterly Volume 17, Issue 4, December 2003, Pages 423–441. 10.1525/maq.2003.17.4.423 14716917

[2] Chandraa M, Ranab P, Chandrac K, Arora V (2019). Tuberculosis - depression syndemic: a public health challenge. Ind J Tuberc.

[3] Prince M, Patel V, Saxena S, Maj M, Maselko J, Phillips MR (2007). No health without mental health. Lancet.

[4] WHO Global Tuberculosis Report, 2018 World health organisation, Geneva. https://www.who.int/tb/publications/global_report/en/ accessed: 09.10.19.

[5] Walker I, Khan A, Khan M, Ayub R, Ghias K, Walley J (2018). Depression among multidrug-resistant tuberculosis patients in Punjab, Pakistan: a large cross-sectional study. Int J Tuberc Lung Dis.

[6] Das M, Isaakidis P, Van den Bergh R (2014). HIV, multidrugresistant TB and depressive symptoms: when three conditions collide. Glob Health Action.

[7] Vega P, Sweetland A, Acha J (2004). Psychiatric issues in the management of patients with multidrug-resistant tuberculosis. Int J Tuberc Lung Dis.

[8] Wu S, Zhang Y, Sun F (2016). Adverse events associated with the treatment of multidrug-resistant tuberculosis: a systematic review and meta-analysis. Am J Ther.

[9] Hwang TJ, Wares DF, Jafarov A, Jakubowiak W, Nunn P, Keshavjee S (2013). Safety of cycloserine and terizidone for the treatment of drug-resistant tuberculosis: a meta-analysis. Int J Tuberc Lung Dis.

[10] Avong YK, Isaakidis P, Hinderaker SG (2015). Doing no harm? Adverse events in a nation-wide cohort of patients with multidrug-resistant tuberculosis in Nigeria. PLoS One.

[11] Thomas BE, Shanmugam P, Malaisamy M (2016). Psycho-Socio-Economic Issues Challenging Multidrug Resistant Tuberculosis Patients: A Systematic Review. PLoS One.

[12] Koyanagi A, Vancampfort D, Carvalho AF, DeVylder J, Haro JM, Pizzol D (2017). Depression comorbid with tuberculosis and its impact on health status: cross-sectional analysis of community-based data from 48 low- and middle-income countries. BMC Med.

[13] Oh KH, Choi H, Kim EJ, Kim HJ, Cho SI. Depression and risk of tuberculosis: a nationwide population-based cohort study. Int J Tuberc Lung Dis. 2017:1;21;7;804–809. 10.5588/ijtld.17.0038 28633706

[14] Walker I, Shi O, Hicks J, Elsey H, Wei X, Menzies D, et al. Analysis of loss to follow up in 4,099 multidrug-resistant pulmonary tuberculosis patients. Eur Respir J. 2019;1800353. 10.1183/13993003.00353-2018 31073080

[15] Cavanaugh JS, Kurbatova E, Alami NN (2016). Evaluation of community-based treatment for drug-resistant tuberculosis in Bangladesh. Tropical Med Int Health.

[16] Sweetland AC, Galea J, Shin SS, Driver C, Dlodlo RA, Karpati A (2019). Integrating tuberculosis and mental health services: global receptivity of national tuberculosis program directors. Int J Tuberc Lung Dis Disease.

[17] WHO Multi-drug Resistant Tuberculosis Guidelines. World Health Organisation. Geneva. 2019 https://www.who.int/tb/publications/2019/consolidated-guidelines-drug-resistant-TB-treatment/en/ Accessed: 12.10.19.

[18] Walker IF, Khanal S, Hicks JP, Lamichhane B, Thapa A, Elsey H (2018). Implementation of a psychosocial support package for people receiving treatment for multidrug-resistant tuberculosis in Nepal: a feasibility and acceptability study. PLoS One.

[19] Creswell J, Plano CV (2018). Designing and conducting mixed methods research.

[20] Malterud K, Siersma V, Guassora A (2016). Sample size in qualitative interview studies: guided by information power. Qual Health Res.

[21] Bronfenbrenner U (1977). Toward an experimental ecology of human development. Am Psychol.

[22] First MB, Spitzer RL, Gibbon M, Williams JB (2002). Structured clinical interview for DSM-IV-TR axis I disorders, research version, patient edition.

[23] Firoz A, Karim M, Alam M, Rahman A, Zaman M (2006). Prevalence, medical care, awareness and attitude towards mental illness in Bangladesh. Bangladesh J Psychiatr.

[24] Uddin MJ, Alam MT, Ahmed HU, Khan NM, Hamid M, Chowdhury WA (2015). Psychiatric morbidity among caregivers of schizophrenia patients a study in tertiary care psychiatric Hospital in Dhaka. J Curr Adv Med Res.

[25] Braun V and Clarke V (2006) Using thematic analysis in psychology, Qual Res Psychol, 3:2, 77–101, DOI: 10.1191/1478088706qp063oa.

[26] Agresti A, Coull BA (1998). Approximate is better than "exact" for interval estimation of binomial proportions. Am Stat.

[27] Dupont WD (2019). Statistical Modelling for biomedical researchers: a simple introduction to the analysis of complex data.

[28] R Core Team (2018). R: a language and environment for statistical computing. R Foundation for statistical computing, Vienna, Austria. URL: https://www.R-project.org/.

[29] Sundar Dorai-Raj (2014). binom: Binomial Confidence Intervals For Several Parameterizations. R package version 1.1–1. URL: https://CRAN.R-project.org/package=binom.

[30] WHO. Depression and Other Common Mental Disorders: Global Health Estimates. Geneva: World Health Organization. 2017 https://apps.who.int/iris/bitstream/handle/10665/254610/WHO-MSD-MER-2017.2-eng.pdf Accessed 10.10.19.

[31] Read JR, Sharpe L, Modini M, Dear BF (2017). Multimorbidity and depression: a systematic review and meta-analysis. J Affect Disord.

[32] Ambaw F, Mayston R, Hanlon C (2017). Burden and presentation of depression among newly diagnosed individuals with TB in primary care settings in Ethiopia. BMC Psychiatry.

[33] Oladimeji O, Ushie BA, Udoh EE (2016). Psychosocial wellbeing of patients with multidrug resistant tuberculosis voluntarily confined to long-term hospitalisation in Nigeria. BMJ Glob Health.

[34] Fitzpatrick C, Floyd K (2012). A systematic review of the cost and cost effectiveness of treatment for multidrug-resistant tuberculosis. Pharmacoeconomics.

[35] Horter S, Stringer B, Reynolds L, Shoaib M, Kasozi S, Casas E (2014). “Home is where the patient is”: a qualitative analysis of a patient-centred model of care for multi-drug resistant tuberculosis. BMC Health Serv Res.

[36] Alderson SL, Foy R, Glidewell L, House AO (2014). Patients understanding of depression associated with chronic physical illness: a qualitative study. BMC Fam Pract.

[37] DiMatteo MR, Lepper HS, Croghan TW (2000). Depression is a risk factor for noncompliance with medical treatment meta-analysis of the effects of anxiety and depression on patient adherence. Arch Intern Med.

